# Development and validation of a novel treatment adherence, satisfaction and knowledge questionnaire (TASK-Q) for adult patients with hypothalamic-pituitary disorders

**DOI:** 10.1007/s11102-024-01425-9

**Published:** 2024-07-08

**Authors:** Sofia Llahana, Kevin C. J. Yuen

**Affiliations:** 1 School of Health & Psychological Sciences, City, University of London, London, UK; 2 Department of Diabetes and Endocrinology, University College London Hospitals National Health Service (NHS) Foundation Trust, London, UK; 3 Departments of Neuroendocrinology and Neurosurgery Barrow Pituitary Center, Barrow Neurological Institute, University of Arizona College of Medicine and Creighton School of Medicine, Phoenix, AZ USA

**Keywords:** Questionnaire, Psychometric scale, Hypothalamic-pituitary disorders, Hypopituitarism, Treatment satisfaction and knowledge, Adherence, Advanced Nurse Practitioner

## Abstract

**Purpose:**

Successful treatment outcomes of adults with hypothalamic-pituitary disorders necessitate the adoption of intricate self-management behaviors, yet current scales for evaluating treatment adherence and satisfaction are inadequate for this patient group. This research introduces a novel treatment adherence, satisfaction and knowledge questionnaire (TASK-Q) developed specifically to identify patients’ unmet needs in better assessing and managing these disorders.

**Methods:**

The study was conducted in three phases: (1) generating items and testing content validity, (2) refining these items through a pilot study, and (3) a main study evaluating the psychometric properties of the TASK-Q scale among 262 adults in a Pituitary Nurse-led Clinic, with 152 (58%) patients completing the questionnaire.

**Results:**

Exploratory factor analysis was used to test the factor structure and construct validity of the TASK-Q, revealing a 22-item scale divided into *Satisfaction and Knowledge* (17 items) and *Adherence* (5 items) subscales, and exhibiting high internal consistency (Cronbach’s α = 0.90). Significant correlations were identified between satisfaction and knowledge (r = 0.67, p < 0.001), satisfaction and adherence (r = 0.23, p = 0.005), and knowledge and adherence (r = 0.43, p < 0.001). Complex treatment regimens, like daily growth hormone injections and adjusting glucocorticoids during illness, negatively affected adherence (p < 0.001).

**Conclusion:**

The TASK-Q is a novel validated scale that can effectively evaluate patients’ perspectives on adherence, knowledge and satisfaction. Our findings highlight the significant impact of Advanced Nurse Practitioners in improving patient self-management behaviors, which likely leads to better treatment outcomes for people with hypothalamic-pituitary disorders.

**Supplementary Information:**

The online version contains supplementary material available at 10.1007/s11102-024-01425-9.

## Introduction

Adults with hypothalamic-pituitary disorders may present incidentally with no overt symptoms or from local tumor mass effects and may exhibit a range of physical, psychological and social symptoms. Management strategies vary from surveillance alone to interventions such as surgery, medication, and/or radiotherapy, depending on the presence of mass effects and/or hyper- or non-functioning lesions. Patients who develop hypopituitarism, whether congenital or acquired, require lifelong hormone replacement therapy. Different therapies require varied dosing schedules: glucocorticoids typically necessitate two to three doses daily, whereas thyroid, estrogen/progesterone, testosterone, and growth hormone are usually administered once daily. Treatment forms also vary, including tablets, transdermal gels, and injections. Additionally, these patients often need multiple concomitant medications, resulting in complex medication regimens due to polypharmacy. The primary objective of hormone replacement therapy is to maintain physiological hormone levels, minimize side effects, and prevent complications associated with under- or over-replacement [1, 2]. Therefore, it is essential for patients to adhere closely to their treatment regimen, understand the reasons behind their medication regimens, and actively engage in treatment planning with their prescribing clinician [2].

A rapid literature review conducted as part of this study aimed to assess the prevalence and determinants of nonadherence to treatment in patients with hypothalamic-pituitary disorders. This review identified a limited number of non-interventional studies, each focusing on adherence to a specific hormone replacement therapy, such as glucocorticoids [3–9], growth hormone [10–12], and testosterone [13–16]. However, these studies did not account for patients who are often on multiple hormone regimens, which poses significant limitation since the overall treatment benefit is unlikely to be achieved unless all hormones are optimally managed [1, 2]. Additionally, studies have shown that patient engagement with their treatment plans and understanding of their hypothalamic-pituitary disorder are critical for optimal care outcomes [17–23]. However, these aspects have not been formally assessed using validated questionnaires tested in this patient population.

Numerous instruments have been developed to assess treatment adherence and satisfaction across various chronic conditions [24–29]. However, none of these instruments specifically address patients with hypothalamic-pituitary disorders. Importantly, they also fail to consider other crucial factors influencing adherence, such as patient awareness of their condition and treatment, side effects, the need for caregivers and/or family members to administer the treatment, and the quality of patient-clinician communication and joint decision-making approach. Research shows that patients who are well-informed and actively engaged in their treatment planning are more likely to adhere to their treatment regimen and effectively manage side effects [27, 30, 31]. Similarly, patient satisfaction with care services and effective clinician-patient communication are critical predictors of adherence, follow-through with treatment plans, and appropriate use of care services [28, 30, 32–34].

The aim of this study was to develop and validate a questionnaire designed to assess medication adherence, treatment knowledge, and patient satisfaction specifically in individuals with hypothalamic-pituitary disorders. This tool aims to identify unmet needs and areas for improvement in patient care and information provision.

## Patients and methods

### Study design

We adopted the *STROBE Statement* [35] and used the *STROBE Checklist* for cross-sectional studies to design the study and report the findings. This quantitative study was conducted in three phases: (1) item generation and content validity testing, (2) a pilot study for item validation and (3) the main study which evaluated the psychometric properties of the TASK-Q scale.

### Item generation and content validity testing

The initial domains for this questionnaire were established after two focus group discussions with a patient advisory board consisting of six individuals with hypothalamic-pituitary disorders. Subsequently, a scoping literature review helped formulate a pool of 65 items to explore the following domains:

- Satisfaction with management of the hypothalamic-pituitary disorders
- Knowledge of treatment and condition
- Adherence to treatment

The MARS-5 scale [36] was adapted, with permission, to assess treatment adherence aspects specific to patients with hypothalamic-pituitary disorders. A thorough review of each item in consultation with the patient advisory board led to the exclusion of statements that were lengthy, irrelevant, ambiguous, double-barreled, or redundant, ultimately reducing the scale to 35 items. To minimize response biases such as acquiescence, affirmation, or agreement, a 5-point Likert scale was used, and statements were either positively or negatively worded [37].

The scale was further reviewed by a panel of six experts (two endocrinologists, one clinical nurse specialist and three patients) who were asked to evaluate each statement for relevance, language, duplication and clarity. As a result, six items were removed and eleven were reworded to enhance clarity. Additionally, all statements were rephrased in the first person to improve readability and personal relevance.

### Ethical considerations and data collection

The study was conducted as part of a service evaluation, confirmed by the UK NHS Health Research Authority and the Organization’s Research and Development Unit to not require formal ethics approval. Data collection involved an anonymized postal survey, including a prepaid return envelope and a participant information leaflet. Returning the completed questionnaire implied consent to participate.

### Pilot study

The draft 29-itemTASK-Q questionnaire, comprising 21 *Satisfaction and Knowledge* and 8 *Adherence* items, was tested for item validation in a pilot survey involving 70 adult patients with hypothalamic-pituitary disorders, achieving a 66% response rate (n = 46; 15 males and 31 females). Within the *Satisfaction and Knowledge* subscale, 2 were removed due to over 20% missing responses and multiple unclear answers. Pearson’s correlation test revealed high correlations (r > 0.700 (p < 0.001) between three pairs of items. Two items measuring the same dimension were merged, and the other four were retained, resulting in a revised 26-item subscale. All 8 items in the *Adherence* subscale were retained.

An expert in linguistics conducted a final review of the item content to enhance language clarity. The scale’s readability was assessed using an on-line calculator from Readability Formulas (https://readabilityformulas.com/), yielding a Flesch-Kincaid grade level of 7.5 (8th grade), a Flesch reading ease score of 64.9 (standard level) and an automated readability index of 6.4 (10–11 years old level). All scores are considered acceptable for an adult with average literacy levels [38].

### Study population

The sample included 262 adult patients with a hypothalamic-pituitary disorders who attended the Pituitary Nurse-led Outpatient Clinic at a large teaching hospital in England during the 12 months prior to the study. The exclusion criteria included: (a) patients under 18 years of age, (b) those not receiving hormone replacement therapy, (c) those unable to speak or understand English, and d) patients who had participated in the earlier pilot survey (N = 70).

### Questionnaire content

*Demographic data* included age, gender, diagnosis, duration of condition, number of endocrine medications and co-morbidities.

*Service provision* for the nurse-led clinic included number of visits, out of clinic contacts, duration of consultation, and free text comments.

*The treatment adherence, satisfaction and knowledge questionnaire (TASK-Q)* consisted of two subscales of 26 items: 1) the first 18-item (5 negatively worded) *Satisfaction and Knowledge* subscale including a 5-point “strongly agree to strongly disagree” Likert scale, and 2) the 8-item (3 negatively worded) *Adherence* subscale including a 5-point “never to always” Likert scale and a 6th “not applicable” point for cases such as taking medication on a monthly/weekly basis rather than daily. High scores indicate high levels of treatment satisfaction, knowledge and adherence.

*The Leeds satisfaction questionnaire (LSQ)*, developed by Hill et al. [39] to evaluate patient satisfaction in a rheumatology nurse-led outpatient clinic, was modified for this study with the author’s permission to evaluate the convergent validity of the TASK-Q. The original LSQ demonstrated high internal consistency (Cronbach’s α = 0.97) and stability (test–retest reliability with Pearson’s r = 0.83; p < 0.001). Similar reliability was observed in the current study (α = 0.96) and a previous study in a nurse-led sexual health clinic (α = 0.94 and test–retest r = 0.95; p < 0.001) [40]. The LSQ comprises 45 items on a 5-point Likert scale ranging from "strongly agree" to "strongly disagree," divided into six subscales: (1) general satisfaction, (2) giving of information, (3) empathy with the patient, (4) technical quality and competence, (5) attitude towards the patient, and (6) access and continuity.

### Data analysis

Data were analyzed using IBM statistical package for social sciences. Negatively worded items were reversed for response uniformity in descriptive statistics. Exploratory factor analysis (EFA) with maximum Likelihood extraction was used to test the factor structure and construct validity of the TASK-Q. Factors were retained based on Cattell’s scree test [41], with required communality values and factor loadings above 0.30. An oblique, direct oblimin rotation allowed correlations between factors [42, 43].

EFA was performed separately for the *Satisfaction and Knowledge* and the *Adherence* subscales due to different scoring and constructs. Internal consistency was assessed using Cronbach’s alpha coefficient test, with acceptable values > 0.7. Pearson’s correlation coefficient explored correlations between variables and factors, and t-test, cross-tabulations and one-way analysis of variance were used to identify differences between groups and to test concurrent validity [43]. Pearson’s correlation coefficient was also used to test convergent validity by assuming positive correlations between the domains of the TASK-Q and LSQ scales. Statistical significance was set at p < 0.05. Thematic content analysis of free-text comments further supported convergent validity.

## Results

### Demographic characteristics and nurse-led service provision

Of the 262 invited participants, 157 questionnaires were returned; 5 were excluded as they had more than 30% missing data giving a final response rate of 58% (N = 152; 68 males and 84 females). Table 1 presents the demographic, diagnosis and treatment characteristics of respondents.

**Table 1:** Demographic and treatment/diagnosis characteristics of patients (N = 152)

Age, years (n = 152)	
Mean ± SD	41.8 ± 15.7
Median (range)	40.0 (19–79)
Sex ratio male/female (n = 152)	68/84
Hypothalamic-pituitary disorder diagnosis (n = 152)	n (%)
Non-functioning pituitary adenoma	46 (30.3)
Prolactinoma	10 (6.6)
Acromegaly	12 (7.9)
Cushing’s syndrome	16 (10.5)
Isolated growth hormone deficiency	14 (9.2)
Idiopathic/congenital hypopituitarism	8 (5.3)
Hypothalamic/pituitary benign brain tumors	27 (17.8)
Hypopituitarism post treatment for ALL or AML Diagnosis not stated	8 (5.3) 11 (7.2)
Duration since diagnosis, years (%) (n = 152)	
Mean ± SD	16.1 ± 11.8
Median (range)	13 (1–45)
> 1	5 (3.3)
1–5	30 (19.7)
6–10	31 (20.4)
11–20	39 (25.6)
< 21	47 (30.7)
Hormone replacement therapy (n = 152)	n (%)
Thyroid hormone	96 (63.2)
Glucocorticoids	69 (45.4)
Gonadal steroids	86 (56.6)
Daily growth hormone	97 (63.8)
Desmopressin	19 (12.5)
Somatostatin analogues	7 (4.6)
Dopamine agonists	5 (3.3)
DHEA	10 (6.6)
Total number of pituitary hormone replacement therapies (n = 152)	n (%)
1	47 (30.9)
2	29 (19.1)
3	32 (21.1)
4	25 (16.4)
5	15 (9.9)
6	4 (2.6)
Patients with concomitant non-endocrine comorbidities	77 (50.7%)

*SD* standard deviation, *ALL* acute lymphoblastic leukemia, *AML* acute myeloid leukemia, *DHEA* dehydroepiandrosterone

Most respondents (n = 93, 61.2%) attended routine endocrine visits every six months. Within the two years prior to the study, 44 (28.9%) respondents had visited the Nurse-led Clinic at least four times, 35 (23%) three times, 42 (27.6%) twice, and 31 (20.4%) at least once. Length of consultation with the endocrine Advanced Nurse Practitioner (ANP) ranged from 10 to 60 min, with the majority (n = 118, 77.7%) lasting 20 to 30 min. Twenty-one respondents (13.8%) required an urgent face-to-face consultation with the endocrine ANP and were seen within three days. Additionally, 53 (34.9%) and 67 (44.1%) respondents used telephone and email, respectively, to contact the endocrine ANP, with email being the preferred communication method for 88 (57.9%) respondents, as video consultations were not available.

### Construct validity and exploratory factor analysis

#### The satisfaction and knowledge subscale

The 18-item subscale was suitable for exploratory factor analysis (EFA), as evidenced by a determinant of 0.00041, surpassing the threshold of 0.00001 to exclude multicollinearity. The Pearson’s correlation matrix indicated no significant high correlations (r > 0.80, p < 0.001). Bartlett’s chi-square test of sphericity confirmed statistical significance (χ^2^ = 1455.62, p < 0.001), and the Kaiser–Meyer–Olkin (KMO) measure of sampling adequacy was 0.898, well above the minimum acceptable value of 0.50 [43]. Cronbach’s α for the subscale was 0.90.

The initial analysis identified four components with eigenvalues above 1, according to Kaiser’s criterion [44]. However, Field notes that this criterion maybe inaccurate for samples under 250 subjects with an average communality below 0.60 [43]. Given that the current study had a sample size of 152 and a mean communality of 0.54, the number of factors was determined using Cattell’s scree test [41]. The scree plot indicated a clear break after the third point, suggesting a 2-factor solution which explained 51.9% of the total variance, with 42.8% attributed to the first factor and 9.1% to the second.

Factor interpretation was facilitated by an oblique, direct oblimin rotation. Except for item 17, all items had loadings above 0.30 (Table 2) and were included in the final scale. The two factors showed moderate correlation (Pearson’s r = 0.65; p < 0.001). The domains were identified as: Factor 1: *Satisfaction with Treatment and Care Service* (α = 0.86), and Factor 2: *Knowledge of Treatment and Condition*” (α = 0.87). Although item 15 loaded similarly on both factors, it was assigned to Factor 2 based on its content. The corrected item-total correlations for the 17-item subscale exceeded 0.30 for both factors, with an overall Cronbach’s α of 0.91 (Table 2).

**Table 2:** Factor loadings, internal consistency and descriptive statistics for the *Satisfaction and Knowledge* subscale (N = 152)

No	Item description	Factor loadings		Reliability		Descriptive statistics	
		F 1	F 2	α	CIC	Mean (SD) range	SUM (SD) range
	Factor 1: 8 items—SAT (satisfaction with treatment and care received)			0.86		4.0^§^ (0.7) 1–5	32.2^#^ (5.5) 8–40
7	I discuss the results of any tests or scans with my endocrine specialist at each clinic visit	0.91	*−0.34*		0.45	4.2 (0.8)	
8	I discuss my treatment plan with my endocrine specialist at each clinic visit	0.80	*0.09*		0.70	4.1 (0.9)	
6	I always receive clear and easy to follow instructions on how to take my medication	0.75	*0.11*		0.33	4.3 (0.8)	
4	I am encouraged to ask questions about my treatment during the clinic visits	0.74	*0.07*		0.73	4.3 (0.9)	
9	I have received information on what to do in special situations such as travelling or illness	0.60	*0.19*		0.80	3.9 (1.1)	
3	My family and/or partner have learned a lot about my condition from my Endocrine Team	0.49	*–0.20*		0.77	3.5 (1.2)	
5	I receive a copy of the letter with treatment details and test results after each clinic	0.41	*−0.09*		0.65	4.3 (0.9)	
14	I have been informed of symptoms I may get if my condition is not well controlled	0.33	*0.28*		0.52	3.4 (1.0)	
17	I do NOT like it that I may have to take hormone treatment for the rest of my life	*0.20*	*0.19*		–	2.6 (1.3)	
	Factor 2: 9 items-KNW (knowledge of treatment and condition)			0.87		3.2^§^ (0.3) 1–5	29.0^#^ (2.8) 9–45
10	I know of the symptoms caused by my endocrine condition if not treated properly	−0.06	0.85		0.71	3.8 (1.1)	
12	I know exactly why I am taking my endocrine medication (hormone treatment)	*0.06*	0.76		0.70	4.0 (1.2)	
11*	I am NOT aware of the side effects that my endocrine treatment can cause	*0.01*	0.72		0.75	2.6 (1.3)	
13*	I do NOT understand the results of by blood tests and what they mean	*0.15*	0.65		0.67	2.5 (1.2)	
2*	I am still unclear about what my condition is and how it is managed	*0.25*	0.58		0.67	2.2 (1.2)	
1	I have been told everything I need to know about my endocrine condition	*0.31*	0.57		0.68	3.9 (1.1)	
18	I can tell from my physical or emotional symptoms if my hormone levels are abnormal	*−0.08*	0.44		0.60	3.6 (1.1)	
16*	I have NOT been informed about the future progression (prognosis) of my condition	*0.11*	0.39		0.43	2.7 (1.2)	
15	I know when my endocrine treatment or hormone replacement is well balanced	*0.40*	0.37		0.33	3.8 (0.9)	
	Eigenvalues	7.70	1.64				
	Variance explained per factor (%)	42.8	9.1				
	Total variance explained (%) for *Satisfaction and Knowledge*	51.9					
	Cronbach’s alpha (α) for *Satisfaction and Knowledge*			0.91			
	Total Mean (SD) for *Satisfaction and Knowledge* (2 factors) subscale, range 1–5					3.6^§^ (0.4)	
	Total SUM (SD) of items for *Satisfaction and Knowledge* (2 factors), range 17–85					61.2 (6.8)^#^	

Item 17 was excluded from total MEAN and SUM and from reliability and CIC analysis

*CIC* corrected item-total correlation, Extraction Method: Maximum Likelihood; Rotation Method: Oblimin with Kaiser Normalization

^*^indicates negatively worded statements; scores were reversed for descriptive analysis

^**§**^Mean score in the Likert scale of 1 (strongly disagree) to 5 (strongly agree)

^**#**^Higher scores indicate better Satisfaction and Knowledge

#### The adherence subscale

The 8-item subscale was suitable for EFA, showing a determinant of 0.212, KMO of 0.611 and statistically significant Bartlett’s test of sphericity (χ2 = 228.78; p < 0.001). The mean communality was 0.49, and Cattell’s scree test [41] suggested a one-factor solution accounting for 30.6% of the total variance. Since only one factor emerged, rotation was not applicable. Only five items had loadings above 0.30 and were retained in the final *Adherence* subscale; items 5, 7 and 8 were excluded. Post-EFA, Cronbach’s α improved to 0.69 from 0.57, and all corrected item-total correlations exceeded 0.30 (Table 3).

**Table 3:** Factor loadings, internal consistency and descriptive statistics for the *Adherence* subscale (N = 152)

N	Item description	Factor loading	Reliability	Descriptive statistics	
			CIC	Mean (SD)	Valid N
	Factor: ADH (adherence to treatment) – 5 items				
4*	I miss over half of the recommended doses of my medication	0.62	0.50	4.9 (0.9)	152
1	I take all my medication on a daily basis	0.61	0.51	3.6 (1.3)	139
6*	I miss most of my medication when I am travelling or away from home	0.61	0.48	4.1 (1.2)	152
2	I take my medication at the recommended dose and time	0.60	0.47	4.0 (1.1)	152
3*	I miss at least one dose of my recommended medication each week	0.38	0.32	4.0 (1.0)	143
5*	I find it inconvenient to take my medication when away from home	*0.29*	–	3.6 (1.3)	152
7	I inform my specialist if I missed any medication prior to my blood test	*0.11*	–	3.2 (1.6)	152
8	I adjust my medication if necessary when I feel ill or unwell	*0.23*	–	3.1 (1.5)	107
	Total variance explained (%)	30.6			
	Cronbach’s alpha (α) for *Adherence* subscale		0.69		
	Total subscale Mean and SD (5 factor items), range 1 – 5			4.1 (0.8)	139
	Total subscale SUM (SD) and Range (5 factor items) range 5 – 25			19.8 (4.1)	

*Items with factor loadings below 0.30 (5, 7, 8) were excluded from total MEAN and SUM and from reliability and CIC analysis*

*CIC* corrected item-total correlation*, Extraction Method: Maximum Likelihood*

^*^Indicates negatively worded statements; scores were reversed for descriptive analysis

^***§***^*Mean score in the Likert scale of 1 (never) to 5 (always) and 6 (not applicable). Cases with “not applicable” responses were excluded from the Descriptive statistics table*

^***#***^*Higher scores indicate better Adherence to treatment*

The revised 22-item TASK-Q scale demonstrated high internal consistency with a Cronbach’s α of 0.90, an increase from 0.85 before EFA.

**Fig. 1:**
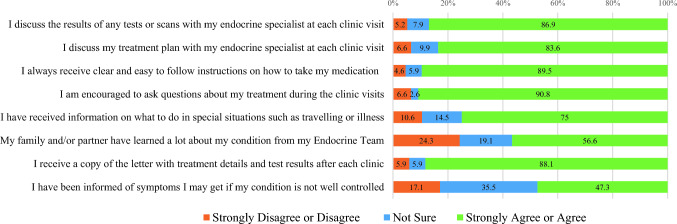
Percentage of responses for items in the *Satisfaction* subscale (N = 152)

**Fig. 2:**
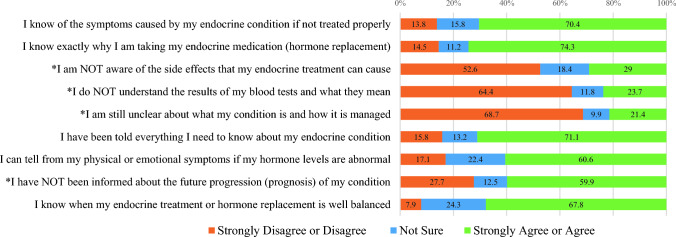
Percentage of responses for items in the *Knowledge* subscale (N = 152)

**Fig. 3:**
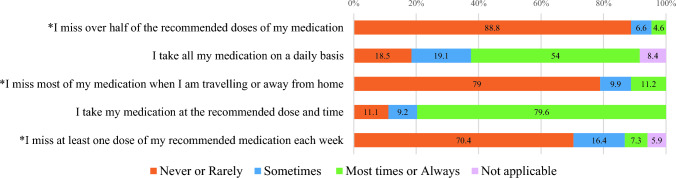
Percentage of responses for items in the *Adherence to treatment* scale (N = 152)

Figures 1, 2, 3 depict the percentage in frequencies of responses for the TASK-Q domains.

Pearson’s product-moment correlation test showed a significant positive relationship between the three factors (domains) of the TASK-Q scale, as follows:

1. r (F1: *satisfaction* and F2: *knowledge*) = 0.67, p < 0.001;
2. r (F1: *satisfaction* and F3: *adherence*) = 0.23, p = 0.005;
3. r (F2: *knowledge* and F3: *adherence*) = 0.43, p < 0.001.

A one-way ANOVA analysis between the mean score of *Knowledge* and *Adherence* items [F(13, 123) = 3.66, p < 0.001] indicates that patients knowledgeable about the management of their condition were more likely to adhere to their treatment regimens and were able to recognize the benefits and necessity of their medication. Additionally, significant correlations were observed between *Satisfaction* items 6, 8, 9, 14 (Table 2) and the mean *Adherence* score [r = 0.272, r = 0.252, r = 0.332, r = 0.250; p < 0.001 respectively]. Further one-way ANOVA analysis confirmed these findings [item 6: F(3, 123) = 3.50; item 8: F(3, 123) = 2.80; item 9: F(3, 123) = 3.26; item 14: F(3, 123) = 2.41; all significant at p < 0.001], suggesting that patients who were well-informed about their medication and potential side effects, and actively involved in their treatment planning, demonstrated better treatment adherence.

#### Criterion-related validity: concurrent and convergent validity

##### Concurrent validity and determinants of nonadherence

A one sample t-test analysis indicated no significant differences in satisfaction and knowledge levels between males and females; however, females reported significantly higher adherence to medication (M = 4.11, SE = 0.95) compared to males (M = 3.82, SE = 0.92) [*t* (150) = −2.16, p = 0.03]. Respondents diagnosed for more than 6 years reported significantly higher levels of satisfaction and knowledge (M = 3.91, SE = 0.06) compared to those diagnosed for less than 6 years (M = 3.43; SE = 0.12) [*t* (150) = −3.80, p < 0.001], though there were no differences in treatment adherence.

A series of crosstab analyses revealed no significant associations between the mean adherence score and individual pituitary replacement therapies, namely levothyroxine, glucocorticoids, estrogen, testosterone, growth hormone, and desmopressin. Similarly, no correlations were found between adherence and diagnosis categories as per Table 1, although the small number of respondents per category may explain this finding. Notably, patients on two or more pituitary replacement therapies reported significantly higher adherence (M = 4.13, SE = 0.07) than those on only one therapy (M = 3.56, SE = 0.14) [*t* (66) = −3.56, p = 0.001]. Cross-tabulation showed that 44.6% (21 of 47) of respondents taking only one medication were on daily growth hormone (GH) injections. Among the 97 respondents on daily GH injections, 24 (24.7%) reported they never or rarely took all their injections daily, 26 (26.8%) missed at least one injection per week, and 23 (23.7%) missed most injections when away from home.

A negative correlation was observed between glucocorticoid replacement therapy and adjusting medication during sick days (r = −0.415, p < 0.001); however, this improved to a positive correlation in patients diagnosed for more than 6 years (r = 0.241, p < 0.05). Cross-tabulation showed that among the 69 patients on glucocorticoid replacement, 16 (23%) rarely or never increased their hydrocortisone dose when unwell, despite 58 (91.6%) of them having been informed about how to adjust their treatment during sick days. Additionally, a lack of awareness about medication side effects correlated with nonadherence (r = −0.421, p < 0.01). Patients informed about potential symptoms of poor condition management and those able to recognize hormone imbalances reported higher treatment adherence levels (r = 0.250, p < 0.01 and r = 0.369, p < 0.01 respectively).

A negative correlation was found between the mean adherence score and the presence of health problems in addition to the endocrine condition (r = −0.205, P = 0.015), suggesting that patients with comorbidities reported impaired adherence to their treatment, likely due to the complexities of polypharmacy. Furthermore, a chi-square test revealed a significant association between the presence of comorbidities and the frequency of attending the endocrine clinic [χ^2^(3) = 11.171, p = 0.011, N = 152], highlighting the increased support needs of these patients. However, no associations were found between the frequency and length of consultations and the treatment satisfaction, knowledge and adherence.

##### Convergent validity (LSQ and free-text analysis)

Strong positive correlations, significant at the p < 0.01 level, were observed between most domains of the TASK-Q and LSQ scales using Pearson’s product-moment correlation test, supporting convergent validity (Table 4). This indicates that patients satisfied with their care tend to report higher levels of treatment satisfaction, knowledge, and adherence.

**Table 4:** Correlations between TASK-Q and LSQ domains (Pearson’s product moment correlation test)

Domain	LSQ-total	LSQ-gsat	LSQ-info	LSQ-emp	LSQ-comp	LSQ-attit	LSQ-cont
TASK-Q total	0.78**	0.66**	0.74**	0.78**	0.68**	0.70**	0.62**
SAT and KNW	0.78**	0.66**	0.75**	0.80**	0.67**	0.70**	0.62**
F1_SAT	0.70**	0.56**	0.70**	0.53**	0.57**	0.59**	0.84**
F2_KNW	0.73**	0.63**	0.68**	0.72**	0.68**	0.69**	0.55**
ADH-1F	0.30**	0.28**	0.24*	0.29**	0.31**	0.30**	0.24*

*TASK-Q* treatment adherence, satisfaction and knowledge questionnaire, *SAT* satisfaction, *KNW* knowledge, *ADH* adherence, *LSQ* Leeds Satisfaction Questionnaire, *LSQ-gsat* general satisfaction, *LSQ-info* giving of information, *LSQ-emp* empathy with the patient, *LSQ-comp* technical quality and competence, *LSQ-attit* attitude towards the patient, *LSQ-cont* access and continuity

^*^p < 0.05, **p < 0.01

Thematic content analysis of the 73 (48% of N = 152) free-text comments further validates the TASK-Q. Almost all comments endorsed the TASK-Q content domains and items. Specifically, 36 (49%) patients noted the endocrine ANP's provision of clear and understandable information, 5 (7%) appreciated the support for their family, 14 (19%) felt empowered and engaged in treatment decision-making, 15 (21%) praised the ANP’s professional competence, and 11 (15%) highlighted the empathy and personal approach of the endocrine ANP. Additionally, 19 (26%) patients emphasized the value of continuity of care in the Nurse-led Clinic and the holistic approach adopted by the endocrine ANP, who regarded patients as individuals rather than medical cases.

## Discussion

This study confirms the complexity of managing hypothalamic-pituitary disorders, emphasizing the importance of comprehensive self-management interventions, consistent treatment adherence, and overall healthcare satisfaction. A key finding is the positive correlation between patient knowledge, treatment adherence, and satisfaction. Patients with a deeper understanding of their condition and treatment were more likely to follow their prescribed regimens, aligning with previous research that shows informed patients generally achieve better health outcomes [8, 12, 14, 15, 17, 23, 45, 46].

Notably, patients diagnosed for over six years showed higher knowledge and satisfaction levels, yet the duration of diagnosis did not influence treatment adherence. Surprisingly, almost 25% of patients on glucocorticoid replacement therapy did not adjust their hydrocortisone dosage when unwell, despite being educated about “sick day rules”. This is a significant observation, corroborated by previous studies [20, 46, 47], highlighting that providing information and increasing knowledge do not necessarily lead to behavior change. There are several possible explanations for the lack of behavior change. Patients might have misconceptions about hydrocortisone or their illness, may deny or fail to recognize worsening symptoms of adrenal insufficiency, may hold fixed beliefs about the necessity of hydrocortisone, or may have concerns about its adverse effects, leading to more negative perceptions of their illness [7, 8]. Furthermore, patients may not have had sufficient experience of recognizing and dealing with situations that require glucocorticoid dose adjustment and are, therefore, not able to act adequately during acute stress, despite having prior knowledge of “sick day rules” [3, 20]. Additional factors, such as different socioeconomic backgrounds, may impact on the patient’s care needs for support in developing and maintaining self-management skills. van der Meij et al. proposed customizing the education to the patient level, especially in patients with lower educational background, with more emphasis on repetitive education and increased involvement of the social network, followed by structural follow-up with repeated education and testing of the practical implementation of this knowledge. Hypothetical situations of stress could also be used to test this knowledge and provide targeted feedback which can lead to behavior change [20].

Our study also revealed ongoing challenges in maintaining high adherence rates, especially among patients with complex, multi-drug regimens. This issue is exacerbated in the management of chronic conditions, where polypharmacy can impact adherence [48–50]. In this study, patients receiving daily GH injections reported lower adherence compared to those on tablet hormone replacements. Prior research linked nonadherence to or discontinuation of GH to dissatisfaction with treatment outcomes, negative beliefs about GH, side effects, and concerns about long-term effects [10–12], underscoring the importance of enhanced communication and support in treatment protocols. However, many other factors can also affect adherence to daily GH injections, including lack of awareness about the condition and treatment benefits, improper use of the GH pen device, injection site pain, payer obstacles such as annual or more frequent authorizations, emotional barriers such as peer pressure, forgetting doses, socioeconomic and family-related barriers such as chaotic households, or frequent travel away from home [31].

### Strategies to enhance self-management and treatment adherence

This study underscores the complexity and personal nature of medication-taking behaviors, highlighting that these are not always under the patient's control. Effective self-management can be promoted by inquiring about the emotional and practical impacts of the condition and treatment on patients [50]. Clinicians should be vigilant for adherence 'red flags' such as a history of missed appointments, and proactively engage patients and their families and caregivers in a cooperative, shared decision-making process to develop “easy-to-follow” treatment plans [51, 52]. Findings from our study concur with earlier evidence suggesting that well-informed patients actively involved in planning their treatment are more likely to adhere to medication regimes and manage side effects effectively [27, 30].

Patient satisfaction and adherence improve when treatment plans consider individual preferences and needs [2]. For instance, frequent clinic visits and added education are necessary for patients starting on new treatments, while well-controlled patients may only need routine annual check-ups. Patients should be supported to recognize suboptimal treatment symptoms and to seek help outside routine visits. The Patient Initiated Follow-Up (PIFU) initiative, introduced by the UK National Health Service (NHS) in 2022, enhances proactive self-management and health outcomes [53]. Our study found that patients with comorbidities had poorer adherence and more regular clinic visits; personalized care, including longer consultations and multidisciplinary input, is necessary for these patients to reduce the number of visits and ensure holistic care.

Clinicians should explain the purpose and options of new treatments clearly, setting realistic expectations about health outcomes. For instance, patients should be informed that noticeable improvements in quality of life (QoL) from GH or testosterone replacement may take at least six months, and not all patients experience these improvements [54]. Addressing concerns about side effects and long term complications is crucial for enhancing adherence. Tools like diaries or QoL questionnaires can help monitor treatment and provide positive feedback [2]. Practical strategies like using visual aids, setting reminders, integrating medication into daily routines, and extending prescription durations can mitigate the challenges of nonadherence [50, 51].

### The role of the advanced nurse practitioner in patient self-management

This study highlights the critical role of the endocrine advanced nurse practitioner (ANP) in supporting patients with their self-management behaviors. Nurse-led clinics provide continuity of care that fosters trusting relationships, which are instrumental in boosting patient satisfaction, a crucial determinant of treatment adherence [50]. Patients who perceived high levels of empathy and professionalism from the endocrine ANP reported better treatment adherence and satisfaction. The findings from both the free text analysis and the TASK-Q emphasize the essential functions of endocrine ANPs in ensuring care continuity, advocacy, family support, and professional competence, all of which contribute to the holistic care delivery. These observations align with prior research indicating that effective communication and empathetic approach significantly enhance patient satisfaction and adherence [30, 33, 34]. Notably, Wickramasuriya et al. [55] reported that over 95% adherence rates were achieved by children monitored at a nurse-led clinic that provided personalized GH treatment initiation.

Moreover, endocrine ANPs are pivotal in tailoring treatments to meet individual patient needs, which are vital for managing chronic conditions such as hypothalamic-pituitary disorders. This study highlighted that nurse-led clinics are particularly effective in monitoring and promoting adherence to complex medication regimes and offer a conducive environment for patients to voice their concerns, thereby improving treatment satisfaction and self-management outcomes. Martinez-Momblan et al. [17] found similar benefits with nurse-led interventions in patients with Cushing’s disease, enhancing patient understanding and engagement.

### Significance of psychometric scales in assessing patient knowledge and satisfaction

Condition-specific validated psychometric scales, such as the TASK-Q developed in this study, are essential for accurately measuring patient knowledge, satisfaction, and treatment adherence particularly in managing chronic conditions like hypothalamic-pituitary disorders. These scales provide reliable patient-reported outcomes that are fundamental for enhancing patient-centered care. The TASK-Q demonstrates high internal consistency as indicated by a strong Cronbach’s α of 0.90. This reliability is crucial for generating trustworthy data that can significantly impact clinical decisions and patient management strategies. By identifying gaps in patient understanding or areas of dissatisfaction, the TASK-Q enables clinicians to tailor self-management interventions that improve treatment adherence and health outcomes. Furthermore, data from scales like the TASK-Q offer empirical evidence that can inform healthcare policies and resource allocation, thereby optimizing healthcare delivery and patient outcomes.

### Methodological considerations and limitations

While this study provides significant insights, the limitations particularly in exploring the implications of polypharmacy and the lack of a broader, multi-centered approach, warrant cautious interpretation of the findings. Further research is needed to test the validity of the TASK-Q scale in varied clinical settings, in addition to nurse-led clinics, and broader demographics to enhance its generalizability. Such studies are vital for benchmarking and defining best practices in the management of patients with hypothalamic-pituitary disorders.

## Conclusion

This study provides valuable insights into the factors that enhance treatment adherence in patients with hypothalamic-pituitary disorders, emphasizing the importance of patient knowledge, satisfaction, and strong therapeutic relationships. These elements contribute to better patient-centered care and can be applied to other chronic diseases that pose similar self-management and adherence challenges.

The study also emphasizes the pivotal role of endocrine ANPs in enhancing self-management in this patient group. ANPs not only address medical needs but also deliver educational, psychological, and holistic support, which are crucial for improving patient health outcomes. The findings highlight the significant benefits of dedicated nurse-led care in improving patient satisfaction, knowledge, and adherence, thereby enhancing overall outcomes for patients with hypothalamic-pituitary disorders.

Moreover, this research demonstrates the value of employing validated psychometric tools, such as the TASK-Q scale, to accurately assess patient-reported outcomes. The TASK-Q facilitates patient engagement by offering insights into patients’ perceptions of their condition and comprehension of their treatment plans. Engaged patients, who are well-informed and actively involved in their care decisions, are more likely to adhere to treatment regimens, leading to improved health outcomes.

## Supplementary Information

Below is the link to the electronic supplementary material.

Supplementary file1 (DOC 100 KB)

## Data Availability

Data can be provided on request by the corresponding author.
