# Correction: Development and validation of a novel treatment adherence, satisfaction and knowledge questionnaire (TASK-Q) for adult patients with hypothalamic-pituitary disorders

**DOI:** 10.1007/s11102-024-01455-3

**Published:** 2024-10-12

**Authors:** Sofia Llahana, Kevin C. J. Yuen

**Affiliations:** 1 School of Health & Psychological Sciences, City, University of London, London, UK; 2 Department of Diabetes and Endocrinology, University College London Hospitals National Health Service (NHS) Foundation Trust, London, UK; 3 Departments of Neuroendocrinology and Neurosurgery Barrow Pituitary Center, Barrow Neurological Institute, University of Arizona College of Medicine and Creighton School of Medicine, Phoenix, AZ USA

**Correction to: Pituitary** 10.1007/s11102-024-01425-9

The original version of this article unfortunately contained an omission in Electronic supplementary material section. During publication, the file including the validated scale from this study was missed to include as supplementary material. Now, the complete supplementary material of this article is presented with this erratum.

## Supplementary Information

Below is the link to the electronic supplementary material.

Supplementary file1 (DOC 100 kb)

Supplementary file1 (PDF 144 kb)

