# Supplementary material for: Development and validation of a novel treatment adherence, satisfaction and knowledge questionnaire (TASK-Q) for adult patients with hypothalamic-pituitary disorders
Source: Pituitary. 2024 Jul 8;27(5):673–84. doi: 10.1007/s11102-024-01425-9 (PMC11513723; doi:10.1007/s11102-024-01425-9)
Supplement: Supplementary file 1 — Supplementary file1 (DOC 100 KB) [file 11102_2024_1425_MOESM1_ESM.doc]

**Supplementary material**

**Figure 1:** Screeplot of the number of components (factors) in the *Satisfaction and Knowledge* questionnaire, showing two breaks at components 3 and 5, suggesting a multidimensional factor solution (extraction method: Maximum Likelihood)


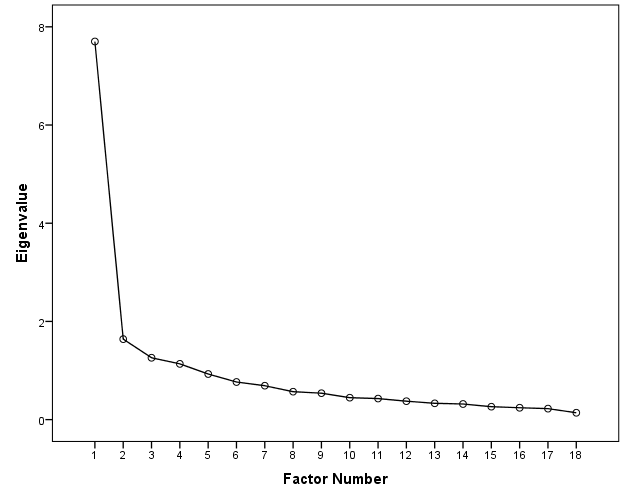


**Table 1: Item statements and descriptive statistics for the *Satisfaction and Knowledge* subscales (N = 152)**

|  | **Item description** | SD  n (%) | D  n (%) | NS  n (%) | A  n (%) | SA  n (%) | Mean (SD) |
| --- | --- | --- | --- | --- | --- | --- | --- |
|  | **Factor 1: 8 items – SAT (satisfaction with treatment and care received)** |  |  |  |  |  |  |
| 3 | My family and/or partner have learned a lot about my condition from my Endocrine Team | 7 (4.6) | 30 (19.7) | 29 (19.1) | 50 (32.9) | 36 (23.7) | 3.5 (1.2) |
| 4 | I am encouraged to ask questions about my treatment during the clinic visits | 3 (2.0) | 7 (4.6) | 4 (2.6) | 62 (40.8) | 76 (50.0) | 4.3 (0.9) |
| 5 | I receive a copy of the letter with treatment details and test results after each clinic | 2 (1.3) | 7 (4.6) | 9 (5.9) | 56 (36.8) | 78 (51.3) | 4.3 (0.9) |
| 6 | I always receive clear and easy to follow instructions on how to take my medication | 2 (1.3) | 5 (3.3) | 9 (5.9) | 65 (42.8) | 71 (46.7) | 4.3 (0.8) |
| 7 | I discuss the results of any tests or scans with my endocrine specialist at each clinic visit | 2 (1.3) | 6 (3.9) | 12 (7.9) | 70 (46.1) | 62 (40.8) | 4.2 (0.8) |
| 8 | I discuss my treatment plan with my endocrine specialist at each clinic visit | 3 (2.0) | 7 (4.6) | 15 (9.9) | 67 (44.1) | 60 (39.5) | 4.1 (0.9) |
| 9 | I have received information on what to do in special situations such as travelling or illness | 8 (5.3) | 8 (5.3) | 22 (14.5) | 63 (41.4) | 51 (33.6) | 3.9 (1.1) |
| 14 | I have been informed of symptoms I may get if my condition is not well controlled | 4 (2.6) | 22 (14.5) | 54 (35.5) | 47 (30.9) | 25 (16.4) | 3.4 (1.0) |
|  | **Factor 2: 9 items – KNW (knowledge and awareness of treatment/condition)** |  |  |  |  |  |  |
| 1 | I have been told everything I need to know about my endocrine condition | 4 (2.6) | 20 (13.2) | 20 (13.2) | 57 (37.5) | 51 (33.6) | 3.9 (1.1) |
| 2* | I am still unclear about what my condition is and how it is managed | 49 (32.2) | 57 (35.5) | 15 (9.9) | 26 (17.1) | 5 (3.3) | 2.2 (1.2) |
| 10 | I know of the symptoms caused by my endocrine condition if not treated properly | 6 (3.9) | 15 (9.9) | 24 (15.8) | 64 (42.1) | 43 (28.3) | 3.8 (1.1) |
| 11* | I am NOT aware of the side effects that my endocrine treatment can cause | 37 (24.3) | 43 (28.3) | 28 (18.4) | 32 (21.1) | 12 (7.9) | 2.6 (1.3) |
| 12 | I know exactly why I am taking my endocrine medication (hormone treatment) | 7 (4.6) | 15 (9.9) | 17 (11.2) | 47 (30.9) | 66 (43.4) | 4.0 (1.2) |
| 13* | I do NOT understand the results of my blood tests and what they mean | 30 (19.7) | 68 (44.7) | 18 (11.8) | 23 (15.1) | 13 (8.6) | 2.5 (1.2) |
| 15 | I know when my endocrine treatment or hormone replacement is well balanced | 1 (0.7) | 11 (7.2) | 37 (24.3) | 71 (46.7) | 32 (21.1) | 3.8 (0.9) |
| 16* | I have NOT been informed about the future progression (prognosis) of my condition | 20 (13.2) | 22 (14.5) | 19 (12.5) | 64 (42.1) | 27 (17.8) | 2.7 (1.2) |
| 17 | I can tell from my physical or emotional symptoms if my hormone levels are abnormal | 11 (7.2) | 15 (9.9) | 34 (22.4) | 60 (39.5) | 32 (21.1) | 3.6 (1.1) |

*SD: Strongly Disagree, D: Disagree, NS: Not Sure, A: Agree, SA: Strongly Agree, SD: Standard Deviation*

**indicates negatively worded statements; scores were reversed for descriptive analysis*

**Table 2 Item statements and descriptive statistics for the *Adherence* subscale** (N = 152)

| N | **Factor: 5 items ADH (adherence to treatment) + 3 dropped items** | Never | Rarely | Sometimes | Most times | Always | N/A | Mean | Valid |
| --- | --- | --- | --- | --- | --- | --- | --- | --- | --- |
|  | Item description | n (%) | n (%) | n (%) | n (%) | n (%) |  | (SD) | N |
| 1 | I take all my medication on a daily basis | 13 (8.6) | 15 (9.9) | 29 (19.1) | 39 (25.7) | 43 (28.3) | 13 (8.6) | 3.6 (1.3) | 139 |
| 2 | I take my medication at the recommended dose and time | 11 (7.2) | 6 (3.9) | 14 (9.2) | 63 (41.4) | 58 (38.2) | 0 | 4.0 (1.1) | 152 |
| 3* | *I miss at least one dose of my recommended medication each week | 55 (36.2) | 52 (34.2) | 25 (16.4) | 7 (4.6) | 4 (2.6) | 9 (5.9) | 4.0 (1.0) | 143 |
| 4* | *I miss over half of the recommended doses of my medication | 114 (75.0) | 21 (13.8) | 10 (6.6) | 3 (2.0) | 4 (2.6) | 0 | 4.6 (0.9) | 152 |
| 5* | *I find it inconvenient to take my medication when away from home | 50 (32.9) | 35 (23.0) | 33 (21.7) | 17 (11.2) | 17 (11.2) | 0 | 3.6 (1.3) | 152 |
| 6* | *I miss most of my medication when I am travelling or away from home | 77 (50.7) | 43 (28.3) | 15 (9.9) | 7 (4.6) | 10 (6.6) | 0 | 4.1 (1.2) | 152 |
| 7 | I inform my specialist if I missed any medication prior to my blood test | 36 (23.7) | 18 (11.8) | 23 (15.1) | 25 (16.4) | 50 (32.9) | 0 | 3.2 (1.6) | 152 |
| 8 | I adjust my medication if necessary when I feel ill or unwell | 27 (17.8) | 13 (8.6) | 17 (11.2) | 23 (15.1) | 27 (17.8) | 45 (29.6) | 3.1 (1.5) | 107 |

**indicates negatively worded statements; scores were reversed for descriptive analysis*

*N/A indicates not applicable responses; these were excluded from mean and SD scores*

*Items 5, 7 and 8 had factor loading <0.3 and were removed from the final Adherence subscale*
