# Supplementary material for: Correction: Development and validation of a novel treatment adherence, satisfaction and knowledge questionnaire (TASK-Q) for adult patients with hypothalamic-pituitary disorders
Source: Pituitary. 2024 Oct 12;27(5):744. doi: 10.1007/s11102-024-01455-3 (PMC11513739; doi:10.1007/s11102-024-01455-3)
Supplement: Supplementary file 2 — Supplementary file1 (PDF 144 kb) [file 11102_2024_1455_MOESM2_ESM.pdf]

TASK-Q, Llahana & Yuen_2024© https://doi.org/10.1007/s11102-024-01425-9
There is no requirement to obtain permission to use the scale, but source the source above must be cited in potential publications. Permission must be obtained from the authors to translate the scale.

Treatment Adherence, Satisfaction & Knowledge Questionnaire (TASK-Q) for Patients with hypothalamic-pituitary disorders

Part A – Knowledge and Satisfaction

This section asks how much you know about your condition and treatment and how satisfied you are with your treatment and the management of your condition. Please state your agreement with each statement below by ticking the relevant box in the table under scorings **from** "***strongly agree***" to "***strongly disagree***".

Please only consider your **pituitary condition and the medication(s)** you take for this when answering these questions.

		strongly agree	agree	not sure	disagree	strongly disagree
1.	I have been told everything I need to know about my endocrine condition					
2.	*I am still unclear about what my condition is and how it is managed					
3.	My family and/or partner have learned a lot about my condition from my Endocrine Team					
4.	I am encouraged to ask questions about my treatment during the clinic visits					
5.	I receive a copy of the letter with treatment details and test results after each clinic					
6.	I always receive clear and easy to follow instructions on how to take my medication					
7.	I discuss the results of any tests or scans with my endocrine specialist at each clinic visit					
8.	I discuss my treatment plan with my endocrine specialist at each clinic visit					
9.	I have received information on what to do in special situations such as travelling or illness					
10.	I know of the symptoms caused by my endocrine condition if not treated properly					
11.	*I am NOT aware of the side effects that my endocrine treatment can cause					

Please continue overleaf

There is no requirement to obtain permission to use the scale, but source the source above must be cited in potential publications. Permission must be obtained from the authors to translate the scale.

Part A (continued)

Please state your agreement with each statement from "***strongly agree***" to **"*strongly disagree*"** by ticking the relevant box in the table below**.**

		**strongly agree**	**agree**	**not sure**	**disagree**	**strongly disagree**
12	I know exactly why I am taking my endocrine medication (hormone replacement)					
13	*I do NOT understand the results of by blood tests and what they mean					
14	I have been informed of symptoms I may get if my condition is not well controlled					
15	I know when my endocrine treatment or hormone replacement is well balanced					
16	*I have NOT been informed about the future progression (prognosis) of my condition					
17	I can tell from my physical or emotional symptoms if my hormone levels are abnormal					

Part B – Adherence to medication

Now, please think of the way you have been taking your medication **in the last 3 to 6 months** when reading the statements in the table below. Please state how often you do each of them by ticking the relevant box across each statement from ***"never"*** to ***"always"***.

		**never**	**rarely**	**some time**	**most of the time**	**always**	**not applicable**
1.	I take all my medication on a daily basis						N/A
2.	I take my medication at the recommended dose and time						N/A
3.	*I miss at least one dose of my recommended medication each week						N/A
4.	*I miss over half of the recommended doses of my medication						N/A
5.	*I miss most of my medication when I am travelling or away from home						N/A

Thank you! Please ensure you have answered all the questions.

There is no requirement to obtain permission to use the scale, but source the source above must be cited in potential publications. Permission must be obtained from the authors to translate the scale.

Scoring instructions and calculating total scores for the TASK-Q subscales

The two subscales (part A and B) should be scored separately as use different Likert scales. The statements for the two subscales are randomly presented, including negatively worded statements, to minimise response bias.

Knowledge and Satisfaction subscale

Each item is given a score based on the Likert scale below:
4 = strongly agree; 3 = agree; 2 = not sure; 1 = disagree; 0 = strongly disagree.

The score for the negatively worded items noted with an asterisk (items 2, 11, 13, 16) should be reversed before calculating the mean and total score, i.e. 4 = 0; 3 = 1; 1 = 3; 0 = 4.

<u>Calculating the *Satisfaction* score</u>

Calculate the total score by adding scores from the following items using the above Likert scale scoring:
Items: 3, 4, 5, 6, 7, 8, 9, 14

Range: 0 – 32 (lowest to highest satisfaction score)

<u>Calculating the *Knowledge* score</u>

Calculate the total score by adding scores from the following items using the above Likert scale scoring:
Items: 1, 2, 10, 11, 12, 13, 15, 16, 17

Range: 0 – 36 (lowest to highest knowledge score).

Add above *Knowledge and Satisfaction* scores for the total subscale score (range 0 – 68).

Adherence subscale

Each item is given a score based on the Likert scale below:
0 = never; 1 = rarely; 2 = sometime; 3 = most of the time; 4; always; 5 = not applicable.

The score for the negatively worded items noted with an asterisk (items 2, 4, 5) should be reversed before calculating the mean and total score, i.e. 4 = 0; 3 = 1; 1 = 3; 0 = 4.

Range: 0 - 20; higher scores indicate better Adherence to treatment. A score of 19 – 20 is considered “excellent adherence”; 16 - 18 as “good adherence”, 13 - 15 as “adequate adherence” and below 14 “poor adherence.

Any responses to point 6 (not applicable) should be removed from the Adherence score, and the range (0 – 20) should be adjusted accordingly, e.g. if one item is scored as “6”, the range should be 0 – 16.
